# Stroke classification and treatment support system artificial intelligence for usefulness of stroke diagnosis

**DOI:** 10.3389/fneur.2023.1295642

**Published:** 2023-12-14

**Authors:** Nobukazu Miyamoto, Yuji Ueno, Kazuo Yamashiro, Kenichiro Hira, Chikage Kijima, Naoki Kitora, Yoshihiko Iwao, Kayo Okuda, Shohei Mishima, Daisuke Takahashi, Kazuto Ono, Mika Asari, Kazuki Miyazaki, Nobutaka Hattori

**Affiliations:** ^1^Department of Neurology, Juntendo University School of Medicine, Tokyo, Japan; ^2^HACARUS INC., Kyoto, Japan; ^3^Ohara Pharmaceutical Co., Ltd., Tokyo, Japan; ^4^PARKINSON Laboratories Co., Ltd., Tokyo, Japan

**Keywords:** stroke, TOAST classification, multimodal artificial intelligence, k-Nearest Neighbor method, leave-one-out cross-validation method, cerebral infarction

## Abstract

**Background and aims:**

It is important to diagnose cerebral infarction at an early stage and select an appropriate treatment method. The number of stroke-trained physicians is unevenly distributed; thus, a shortage of specialists is a major problem in some regions. In this retrospective design study, we tested whether an artificial intelligence (AI) we built using computer-aided detection/diagnosis may help medical physicians to classify stroke for the appropriate treatment.

**Methods:**

To build the Stroke Classification and Treatment Support System AI, the clinical data of 231 hospitalized patients with ischemic stroke from January 2016 to December 2017 were used for training the AI. To verify the diagnostic accuracy, 151 patients who were admitted for stroke between January 2018 and December 2018 were also enrolled.

**Results:**

By utilizing multimodal data, such as DWI and ADC map images, as well as patient examination data, we were able to construct an AI that can explain the analysis results with a small amount of training data. Furthermore, the AI was able to classify with high accuracy (Cohort 1, evaluation data 88.7%; Cohort 2, validation data 86.1%).

**Conclusion:**

In recent years, the treatment options for cerebral infarction have increased in number and complexity, making it even more important to provide appropriate treatment according to the initial diagnosis. This system could be used for initial treatment to automatically diagnose and classify strokes in hospitals where stroke-trained physicians are not available and improve the prognosis of cerebral infarction.

## Highlight

- SCTSS-AI classifies strokes with over 85% accuracy, aiding in treatment decisions.
- Utilizing multimodal data, the AI provides explanations and improves prognosis of cerebral infarction.
- The system addresses the shortage of stroke experts, enabling automatic diagnosis and classification.

## Introduction

Cerebrovascular disease, commonly referred to as stroke, is a leading cause of death and chronic disability on a global scale (1–3). Approximately 80% of strokes are caused by cerebral ischemia (4). In addition, energy depletion and cell death can cause ischemic brain injury (5). These injuries lead to functional impairment of the injured neurons, leading to severe long-term disability. In the initial diagnosis, brain imaging techniques, such as computed tomography (CT) and magnetic resonance imaging (MRI), to detect tissue necrotic areas of cerebral infarction are important tools for ischemic stroke assessment (6).

Treatment of ischemic stroke includes intravenous thrombolysis, intra-arterial therapy, and mechanical revascularization (7). Although it is possible to diagnose stroke without being a stroke specialist, stroke-trained physicians classify stroke severity using knowledge of how the physiology of different stroke types is reflected in image textures (8). However, manual image analysis is labor intensive (8) and prone to inter- and intra-operator variability (9, 10). Furthermore, expert analysis is limited by the number and areas where specialists practice (11), which results in increased diagnostic costs. Automatic lesion identification and subsequent stroke severity classification can significantly reduce drawing time and accurately detect lesions (11). The development of computer-aided detection and diagnostic systems based on the automatic detection of post-stroke brain lesions is an active research field. In such studies, research is being conducted to construct an automated stroke severity classification system using either CT or MRI. Both methods yield a graphical representation of the human brain containing distinct image objects. Identifying such objects through image segmentation is an important step in extracting diagnostically important information. CT is faster and less expensive and more widely used globally than MRI. However, MRI is suitable for constructing an automated stroke severity classification system because MRI is much more sensitive for acute ischemic lesions than CT (12) and MRI scans can be enhanced by adding functional information to the anatomical data to form diffusion-weighted images (DWI).

There are regional disparities in the number of physicians who can diagnose stroke accurately globally. In Japan, there are many stroke-trained physicians in urban areas; however, there are fewer in rural hospitals. Thus, initial stroke treatment is provided by general physicians who are not trained in stroke care (13). To solve this problem, the Japan Stroke Association has provided guidelines on “drip-and-ship treatment,” but this only increases the burden on urban stroke-trained physicians (14). In stroke treatment, it is important to classify the acute phase of stroke and treat patients according to the stroke classification, even in environments where mechanical thrombectomy and intravenous thrombolysis are not available (15). However, differences in functional prognosis have been reported between patients treated by stroke specialists and those treated by general physicians (16). We aimed to develop an artificial intelligence (AI)-based stroke diagnosis aid system using MRI to automatically diagnose and classify strokes in hospitals where stroke-trained physicians are not available, and to link this to initial medical care.

## Patients and methods

### Patients

**Figure 1:**
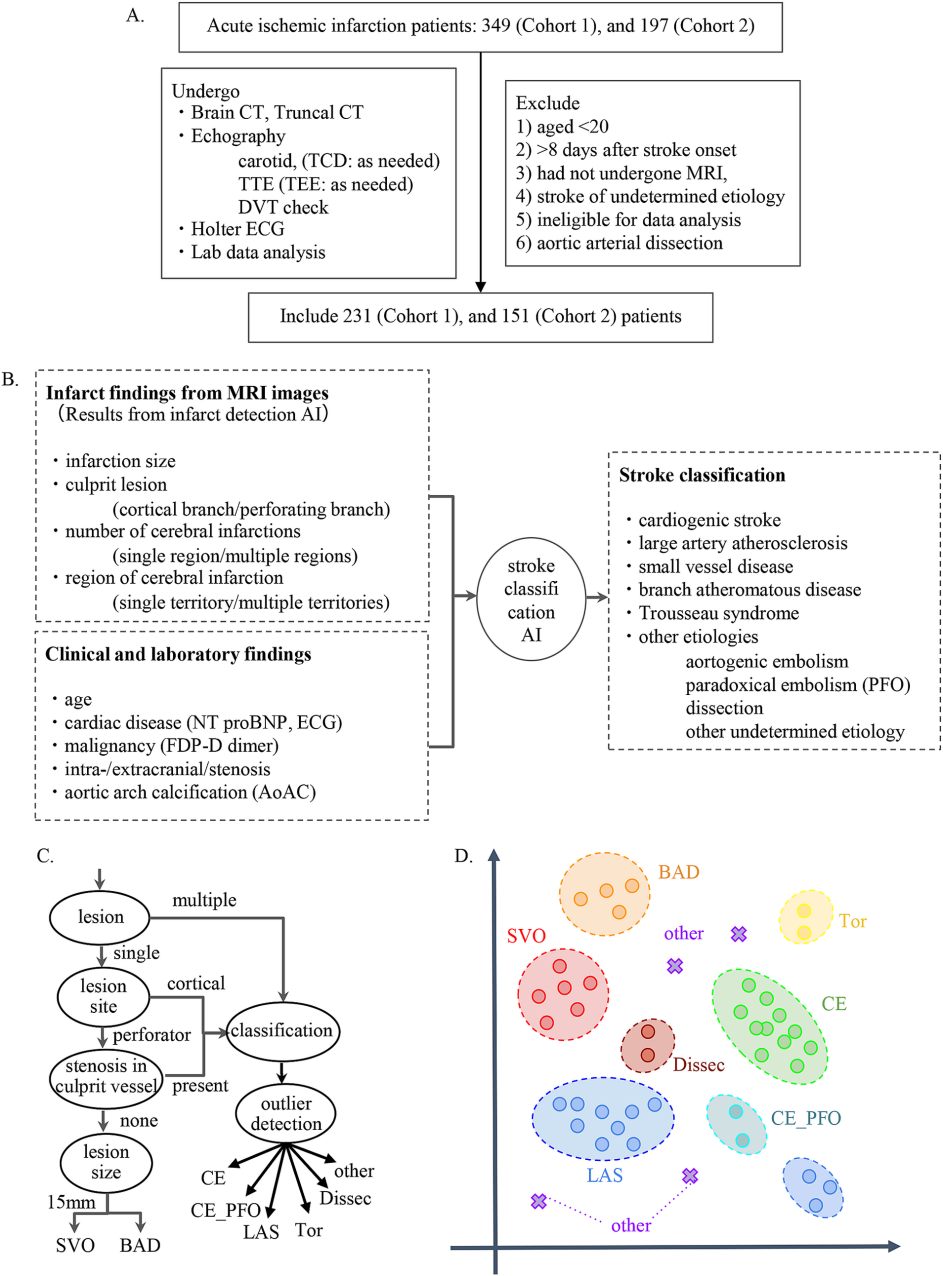
**(A)** Flow chart describing enrollment of patients with stroke in the present study. **(B)** Flowchart for establishment of stroke classification AI. **(C)** Decision flow for developing stroke classification AI. **(D)** k-Nearest Neighbor method. The k-Nearest Neighbor classification algorithm was used to classify SVO, BAD, LAS, and CE. For the other determined etiology, a method combining the concept of abnormality detection was used.

We developed a Stroke Classification and Treatment Support System AI (SCTSS-AI) equipped with the infarct detection AI and the stroke classification AI for cerebral infarction. The development was approved by the Human Ethics Review Committee of Juntendo University School of Medicine (E22-0028). The stroke classification AI was established using the medical records and MRI data of Cohort 1, who were admitted to Juntendo University Hospital’s Neurology Department between January 2016 and December 2017 for cerebral infarction or developed cerebral infarction while admitted and were treated at the Neurology Department (Figure 1A). The infarct detection AI was trained primarily using MRI data from patients in Cohort 1 with the three main types of Trial of Org 10,172 in Acute Stroke Treatment (TOAST) classification. To confirm the accuracy of SCTSS-AI, we used another data set provided from Cohort 2, who were treated at the same institution as Cohort 1 for stroke between January 2018 and December 2018 (Figure 1A).

The exclusion criteria for both cohorts were as follows: 1) patients aged <20 years at stroke onset, 2) patients with stroke >8 days after stroke onset, 3) patients who had not undergone MRI, 4) patients with stroke of undetermined etiology (negative evaluation and two or more causes identified), 5) cases diagnosed with aortic arterial dissection, and 6) patients who were judged by three stroke experts to be ineligible for data analysis. The diagnostic results and treatment methods provided by the system were constructed in accordance with the Japan Stroke Treatment Guidelines 2021 (14) and in the final evaluation, training data and test data were completely separated to evaluate the generalization performance of the system.

### Collected data set

We extracted the following information from the medical records of each patient to establish SCTSS-AI: 1) demographic data; 2) vital signs at presentation and laboratory findings including ECG, fibrin/fibrinogen degradation products [FDP] D dimer, brain natriuretic peptide [BNP], N-terminal pro-BNP [NT-proBNP], estimated glomerular filtration rate, and high-sensitivity C-reactive protein on admission; 3) medications taken upon admission, with particular attention paid to anti-platelets, anti-coagulants, anti-hypertensives, and statins; 4) vascular risk factors for stroke, such as hypertension (HT; systolic blood pressure [BP] > 140 mmHg, diastolic BP > 90 mmHg, or drug treatment for HT), dyslipidemia (DL; defined as low-density lipoprotein [LDL] cholesterol level of >140 mg/dL, high-density lipoprotein [HDL]-cholesterol level of <40 mg/dL, triglyceride [TG] level of >149 mg/dL, or drug treatment for DL), diabetes mellitus (DM; defined as glycated hemoglobin level of >6.4%, or drug treatment for DM), a cardioembolic source according to TOAST classification (17), transient ischemic attack, and smoking history (as reported by the patient and their family); 5) stroke mechanism according to TOAST criteria (17); and 6) baseline National Institutes of Health Stroke Scale (NIHSS) score (18), as recorded by stroke-trained neurologists that were certified in the application of the NIHSS, on admission. Brain CT/MRI and electrocardiography were performed in all patients, and we diagnosed brain infarction by focal hyper-intensity that was judged not attributable to normal anisotropic diffusion or magnetic susceptibility artifact.

### Establishment of infarct detection AI

#### Adjustment of MRI images

We constructed the infarct detection AI that was used to derive the features that determine the stroke classification using MRI from a variety of different resolutions and manufacturers to assess patients who were initially suspected of stroke between 6 h and 7 days from the onset time as training data. The process of correcting the signal values of the MRI images based on the positions of the peaks was performed. To correct for differences in image orientation and position, we used image processing to measure the orientation of the head and perform rotation correction to adjust the tilt and to correct for differences in imaging range in the slice direction (Z-axis) and brain size, and used the Dynamic Time Warping technique to correct the Z-axis position (19). Variations in images among cases were corrected.

#### Identification of features

**Figure 2:**
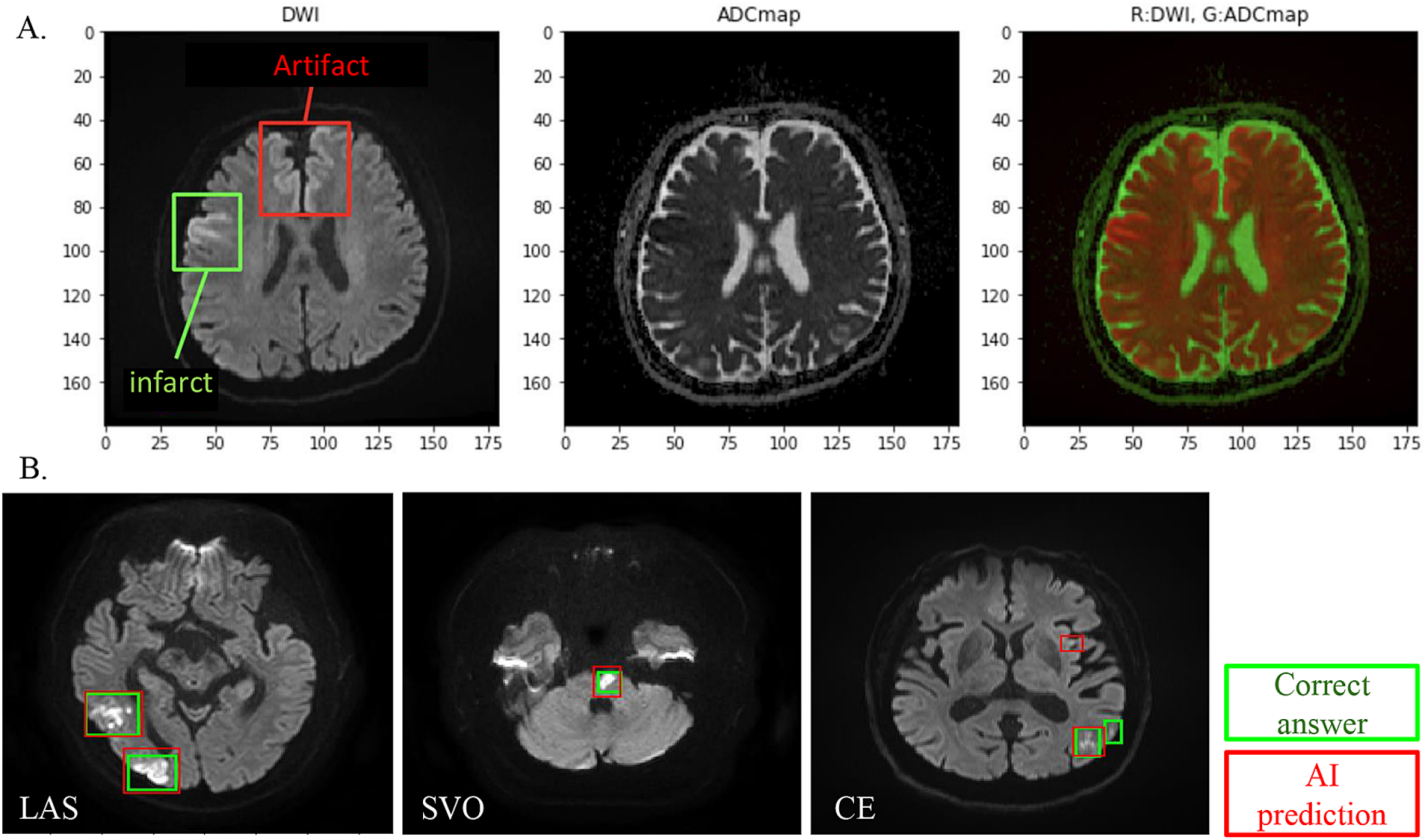
**(A)** DWI and ADC combined analysis method for infarct detection AI visualizes and evaluates the difference between DWI high-intensity area and ADC low-intensity area, except for symmetrical high-signal areas and areas where artifacts are likely to appear. **(B)** Infarct detection AI overview. One hundred cases were studied, and 18 cases were evaluated. In infarct lesion units, the sensitivity (recall) was 80% and the match rate (lesion-by-lesion evaluation) was 84%.

Using the corrected image data, the infarct detection AI derives the following features and provides them to SCTSS-AI. The stroke classification AI is designed to diagnose cerebral infarction based on TOAST classification and to propose treatment methods (Figure 1B). Infarct-related features were as follows: (i) size (< 1.5 cm/1.5 cm or larger), (ii) culprit lesion (cortical branch/perforating branch as the preferred site of small vessel occlusion [SVO]/branch atheromatous disease [BAD]), (iii) number of cerebral infarctions (single region/multiple regions), (iv) region of cerebral infarction (single territory/multiple territories), (v) presence of intracranial stenosis, (vi) presence of carotid artery stenosis, and (vii) presence or absence of cardiac disease as a risk factor for embolic source (including NT-proBNP). Of these, (i)-(iv) were extracted using infarct detection AI. The infarct detection AI was built using DWI and apparent diffusion coefficient (ADC) mapping, which are used to distinguish between infarcts and artifacts. The design of the features to be used as machine learning input was based on domain knowledge about the difference between infarcts and artifacts. The 3D positional and symmetry information of candidate pixels were used as features in the construction of the infarct detection AI, based on the artifacts tending to occur at specific locations and symmetrically. To distinguish between the feature of high signal at the infarction point of DWI, and cases where the high signal is not due to the infarction point but to the effect of T2 shine through, the pixel value of the ADC map and the amount of information of surrounding pixel values were also used as feature values (Figure 2A). Designing input features that leverage domain knowledge is difficult to incorporate into deep learning-based machine learning and is one of the major differences between the AI that we built and deep learning-based AI.

#### Annotation MRI images

To build the infarct detection AI to derive the above features, stroke experts with more than 10 years of experience annotated the MRI dataset as training data.

### Construction of stroke classification AI

SCTSS-AI was designed so that the infarct detection AI and the stroke classification AI work in tandem to classify stroke. The infarct detection AI extracts the features from the MRI data, and the stroke classification AI combines infarct-related features and stroke classification-related features from the medical records to make a diagnosis.

#### Selection of features

**Figure 3:**
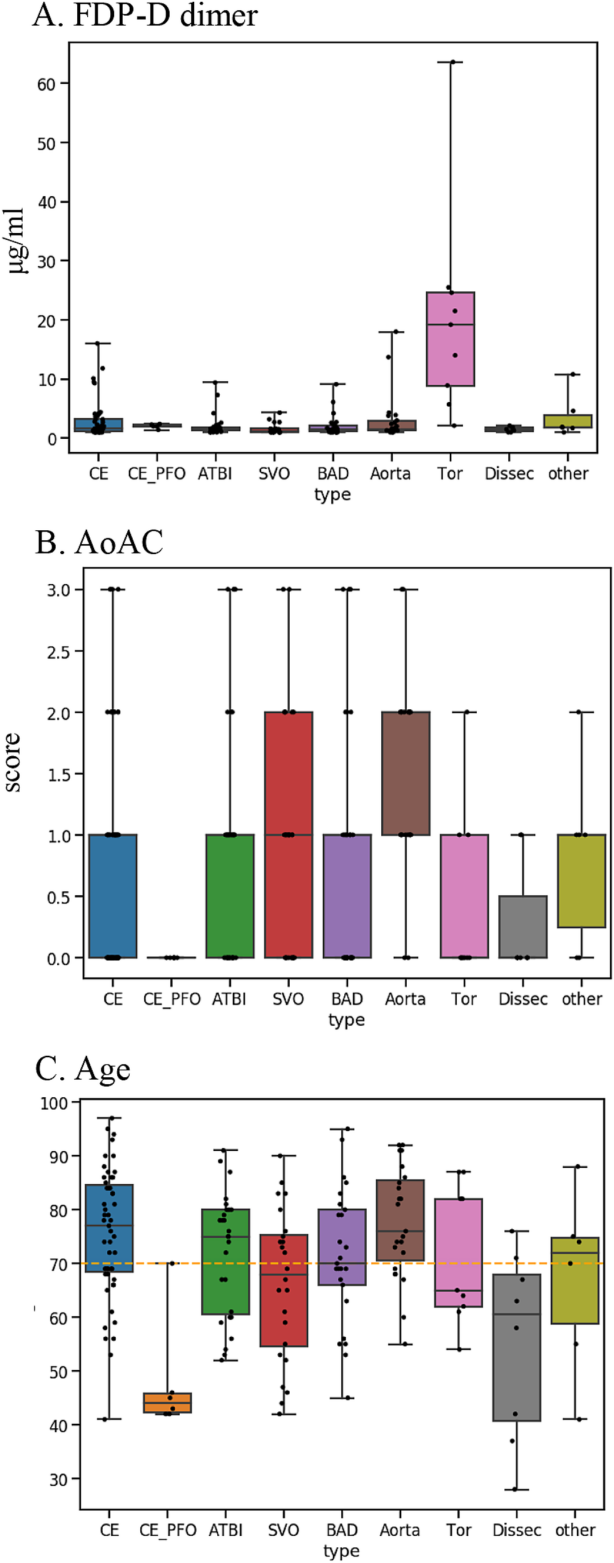
Key features used for disease typing AI: **(A)** D-dimer. **(B)** AoAC [Shimada et al. (17)]. **(C)** Age. AoAC increased in aortogenic embolism, and patients with CE-PFO/dissection were younger than other etiologies. FDP-D dimer was higher in patients with Trousseau syndrome.

The stroke classification AI was constructed using the patients’ background, medical history, and clinical and laboratory findings used to classify stroke. The following additional features related to stroke classification were selected by 231 of the cases provided by Juntendo Hospital (Figure 1A): (viii) malignancy (treated/not treated), (ix) D-dimer, (x) grade of aortic arch calcification (AoAC) by chest X-ray (grade 0–3), and (xi) age. Features were selected based on TOAST criteria and our domain knowledge. These features were reported in previous studies and also confirmed in our analysis. It was noted that the blood fibrin degradation products, such as D-dimer, tend to be higher in Trousseau syndrome than in other stroke types due to hypercoagulability caused by malignancy, that AoAC tends to be higher in aortic primary cerebral embolism than in other stroke types (20), and that cardiogenic cerebral embolism due to patent foramen ovale (PFO) and arterial dissection are common stroke types in young patients (Figure 3).

#### Algorithm design

We designed an algorithm that makes inferences if part of the information is missing, as some tests necessary for diagnosis are not performed immediately after MRI in the clinical setting. The AI executes an appropriate model corresponding to features with missing values. The algorithm for diagnosis of cerebral infarction was constructed based on the TOAST classification approach (Figure 1C). This algorithm was based on the long-used algorithm of Lee et al. (21), which was modified to incorporate TOAST classification from imaging. In addition, we employed the k-Nearest Neighbor (kNN) method to classify stroke other than Others, and combined kNN with anomaly detection for Others (Figure 1D).

#### Evaluation methods & statistical analysis methods

The performance of the AI constructed from the 231 cases of Cohort 1 was assessed by the Leave One Out Cross Validation (LOOCV) method (22). Out of the 231 cases in Cohort 1, one case was extracted as the data for evaluation, and stroke was classified using the stroke classification AI trained with the remaining data and compared with the stroke classification determined by the stroke experts. This evaluation was repeated until all 231 cases were used as data for evaluation, and the stroke classification AI was evaluated. Final accuracy of the stroke classification AI with verified performance using LOOCV was evaluated using 151 independent cases from Cohort 1 (Cohort 2).

All data analysis, including image processing and feature extraction of the infarct detection AI, training and evaluation of the stroke classification AI, and visualization were performed under the Python 3.7 environment. The performance metrics used in the evaluation of the stroke classification AI were: accuracy sensitivity, precision, and *F* value expressed by the following formulas:


$$TP+FN=\mathrm{Patient}\;\mathrm{numbe}r_{\mathrm{stype}}$$

$$TP+FP=\mathrm{Predicted}\;\mathrm{number}\;\mathrm{of}\;\mathrm{case}s_{\mathrm{stype}}$$

$$TP=\mathrm{Number}\;\mathrm{of}\;\mathrm{correct}\;\mathrm{case}s_{\mathrm{stype}}$$

$$\begin{array}{l} \mathrm{sensitivit}y_{\mathrm{stype}}=\frac{\mathrm{Number}\;\mathrm{of}\;\mathrm{correct}\;\mathrm{case}s_{\mathrm{stype}}}{\mathrm{Patient}\;\mathrm{numbe}r_{\mathrm{stype}}},\\ \mathrm{precisio}n_{\mathrm{stype}}=\frac{\mathrm{Number}\;\mathrm{of}\;\mathrm{correct}\;\mathrm{case}s_{\mathrm{stype}}}{\mathrm{Predicted}\;\mathrm{number}\;\mathrm{of}\;\mathrm{case}s_{\mathrm{stype}}} \end{array}$$

$$\begin{array}{l} F\;\mathrm{valu}e_{\mathrm{stype}}=\frac{2^{*}\mathrm{sensitivit}{y_{\mathrm{stype}}}^{*}\mathrm{precisio}n_{\mathrm{stype}}}{\mathrm{sensitivit}y_{\mathrm{stype}}+\mathrm{precisio}n_{\mathrm{stype}}},\\ \mathrm{accuracy}=\frac{\sum_{\mathrm{stype}}\mathrm{Number}\;\mathrm{of}\;\mathrm{correct}\;\mathrm{case}s_{\mathrm{stype}}}{\sum_{\mathrm{stype}}\mathrm{Patient}\;\mathrm{numbe}r_{\mathrm{stype}}} \end{array}$$

where TP is True Positives, FN is False Negatives, FP is False Positives, and stype is subtype. Sensitivity, precision, and *F* value were calculated for each subtype. Accuracy was calculated for all patients. Sensitivity indicates how many patients with a subtype were actually detected as patients. Precision indicates how correct the predicted result was. F value is an integrated value of sensitivity and Precision and can be evaluated considering their trade-off. Accuracy indicates how many patients overall were predicted as having the correct disease type.

### Statistical analysis

The data were analyzed with SPSS 29.0 (SAS Institute Inc., Cary, NC). Data are expressed as mean ± standard deviation values for continuous variables. All statistical analyzes were performed using χ^2^ test for categorical variables, t-test for parametric analyzes. *p*-values of < 0.05 were considered significant.

## Results

As cohort 1, 231 from 278 people, 151 from 197 people as cohort 2 were enrolled. Background factors and examination data of cohort 1 and 2 patients revealed no difference between cohort 1 and 2, except diastolic blood pressure, heart rate, stroke classification, blood sugar, HbA1c, triglyceride, eGFR on arrival (Table 1).

**Table 1:** Background factors, stroke classification and examination data.

	Cohort 1 (231)		Cohort 2 (151)		*p*-value
	*N*	%	*N*	%	
Sex (male)	154	66.7	94	62.2	0.377
Age	69.4 ± 14.8		70.9 ± 14.2		0.172
Body height (cm)	161.4 ± 9.8		160.4 ± 9.3		0.250
Body weight (kg)	60.8 ± 13.3		59.0 ± 14.0		0.107
BMI (kg/m^2^)	23.2 ± 3.8		22.6 ± 3.72		**0.047**
Systolic blood pressure on arrival (mmHg)	150.3 ± 29.8		153.3 ± 28.2		0.220
Diastolic blood pressure on arrival (mmHg)	82.2 ± 17.7		85.4 ± 18.0		**0.049**
Heart rate on arrival (/min)	76.1 ± 15.4		79.8 ± 14.9		**0.050**
Smoking habit	64	27.7	29	19.2	0.058
HT	150	64.9	111	73.5	0.078
DM	53	22.9	58	38.4	**0.001**
DL	79	34.1	101	66.8	**<0.001**
Ischemic heart disease	22	9.5	14	9.2	0.934
Af	39	16.8	37	24.5	0.068
Active Malignancy	18	7.79	13	8.6	0.775
*Stroke classification*					**0.005**
SVO	25	10.8	19	12.5	
BAD	28	12.1	14	9.2	
LAS	29	12.5	25	16.5	
CE	51	22.0	52	34.4	
CE-PFO	7	3.0	1	0.6	
aortogenic embolism	40	17.3	18	11.9	
Trousseau syndrome	9	3.8	9	5.9	
Dissection	15	6.4	8	5.2	
other	27	11.6	5	3.3	
*Laboratory data on arrival*					
WBC (/μL)	7,596 ± 3,490		7,194 ± 2,919		0.126
PT-INR	1.08 ± 0.22		1.10 ± 0.19		0.305
FDP-D dimer (μg/mL)	3.24 ± 7.08		4.15 ± 7.29		0.118
Blood sugar (mg/dL)	124.5 ± 49.2		138 ± 65.3		**0.009**
HbA1c (%)	6.15 ± 1.05		6.57 ± 1.71		**0.002**
LDL (mg/dL)	116.4 ± 36.8		116.5 ± 49.3		0.496
HDL (mg/dL)	51.3 ± 15.0		50.3 ± 16.5		0.270
TG (mg/dL)	120.6 ± 72.6		143.5 ± 104.2		**0.009**
UA (mg/dL)	5.50 ± 1.44		5.48 ± 1.70		0.439
Cre (mg/dL)	0.945 ± 0.984		1.063 ± 1.144		0.149
eGFR (mL/min/1.73 m^2^)	75.53 ± 30.72		65.02 ± 26.13		**<0.001**
hsCRP (mg/dL)	1.262 ± 3.277		1.598 ± 3.755		0.186
NT-proBNP (pg/dL)	1051.88 ± 2658.45		1302.85 ± 4150.58		0.266

Bolded numbers indicate *p* < 0.05 compare to cohort1 and cohort2. CE, cardiogenic embolism; CE-PFO, cerebral embolization through a patent foramen ovale (paradoxical embolism); LAS, large artery atherosclerosis; SVO, small vessel occlusion; BAD, branch atheromatous disease.

### AI for infarct detection

We evaluated the infarct detection AI that determines the presence of absence of infarction using a machine learning algorithm called gradient boosting decision tree using 18 cases in Cohort 1 (SVO: 4 cases; cardiogenic embolism [CE]: 10 cases; large artery atherosclerosis [LAS]: 4). We found sensitivity (reproducibility: the percentage of infarcts that the AI could detect as infarcts out of those that were actually infarcts) of 80 and 84% (lesion-by-lesion evaluation). Thus, our infarct detection AI had a sensitivity (recall) of 80%, with few undetected infarcts and few false detections of infarcts (Figure 2B).

### AI for stroke classification

The results of the evaluation of the stroke classification AI trained using features of 231 cases: SVO/BAD: 53 cases, CE: 51 cases, LAS: 29 cases, Trousseau syndrome: 9 cases, and Others, including aortogenic embolism, paradoxical embolism, and other undetermined etiology (embolic stroke of undetermined sources and cerebral artery dissection [ESUS+D]: 89 cases) in Cohort 1 using the LOOCV method showed an 88.7% correct rate (Tables 2, 3).

**Table 2:** Result for stroke classification AI. Matching table for each stroke type: CE, LAS, SVO/BAD, and ESUS+D in Cohort 1.

		Precision subtype by AI				
		CE	LAS	SVO/BAD	Tro	ESUS+D
Manually assigned stroke subtype	CE	**49**	0	1	1	0
	LAS	0	**29**	0	0	0
	SVO/BAD	0	0	**52**	0	1
	Tro	0	1	1	**4**	3
	ESUS+D	2	5	9	2	**71**

Bold numbers indicate the number of each case correctly answered by the AI. Tro, Trousseau syndrome; ESUS + D, embolic stroke of undetermined sources + cerebral artery dissection; TP, true positives; FN, false negatives; FP, false positives.

**Table 3:** Result for stroke classification AI. Precision rate for each stroke classification.

Stroke classification	Patient number TP + FN	Predicted number of cases TP + FP	Number of correct cases TP	Sensitivity TP/(TP + FN)	Precision TP/(TP + FP)	*F* value
CE	51	51	49	96%	96%	0.96
LAS	29	35	29	100%	83%	0.91
SVO/BAD	53	63	52	98%	83%	0.90
Tro	9	7	4	44%	57%	0.50
ESUS+D	89	75	71	80%	95%	0.87

Tro, Trousseau syndrome; ESUS + D, embolic stroke of undetermined sources + cerebral artery dissection; TP, true positives; FN, false negatives; FP, false positives.

In the present evaluation, high accuracy was obtained for each stroke classification, especially for CE, LAS, and BAD/SVO; however, when attempting to predict BAD/SVO and ESUS+D more finely, the accuracy rate was lower (correct rate of 76.6%, Tables 4, 5). Because SVO/BAD is diagnosed within 1–3 days of the onset of the disease, it is difficult to distinguish SVO/BAD using the information available in the emergency room. Furthermore, it is impossible to diagnose paradoxical embolism unless transesophageal echocardiography is performed. On the other hand, if the patient has been treated for carcinoma, and the FDP D-dimer is elevated (Figure 3A), Trousseau syndrome could be allowed for differentiation. Another problem is that accurate reading of magnetic resonance angiography (MRA) is often difficult for non-stroke physicians. In the acute phase of treatment, the classification into CE, LAS, BAD/SVO, Trousseau syndrome, and ESUS+D may be more practical.

**Table 4:** Result for stroke classification AI. Matching table for each stroke type.

		Precision subtype by AI								
		CE	CE-PFO	LAS	SVO	BAD	Aorta	Tro	Dissec	other
Classification from physician	CE	**49**	0	0	0	1	0	1	0	0
	CE-PFO	0	**0**	0	0	1	1	0	0	5
	LAS	0	0	**29**	0	0	0	0	0	0
	SVO	0	0	0	**25**	0	0	0	0	0
	BAD	0	0	0	0	**27**	1	0	0	0
	Aorto	0	0	2	1	0	**30**	2	0	5
	Tro	0	0	1	0	1	3	**4**	0	0
	Dissec	2	0	2	4	1	4	0	**1**	1
	Other	0	0	1	2	0	10	0	2	**12**

Bolded numbers indicate the number of each case correctly answered by the AI. CE, cardiogenic embolism; CE-PFO, cerebral embolization through a patent foramen ovale (paradoxical embolism); LAS, large artery atherosclerosis; SVO, small vessel occlusion; BAD, branch atheromatous disease; Aorta, aortogenic embolism; Tro, Trousseau; Dissec, cerebral artery dissection; Other, undetermined etiology; TP, true positives; FN, false negatives; FP, false positives.

**Table 5:** Result for stroke classification AI. Precision rate for each stroke classification.

Stroke classification	Patient number TP + FN	Predicted number of cases TP + FP	Number of correct cases TP	Sensitivity TP/(TP + FN)	Precision TP/(TP + FP)	*F* value
CE	51	51	49	96%	96%	0.96
CE-PFO	7	0	0	0%	–	–
LAS	29	35	29	100%	83%	0.91
SVO	25	32	25	100%	78%	0.88
BAD	28	31	27	96%	87%	0.92
Aorto	40	49	30	75%	61%	0.67
Tro	9	7	4	44%	57%	0.50
Dissec	15	3	1	7%	33%	0.11
Other	27	23	12	44%	52%	0.48

CE, cardiogenic embolism; CE-PFO, cerebral embolization through a patent foramen ovale (paradoxical embolism); LAS, large artery atherosclerosis; SVO, small vessel occlusion; BAD, branch atheromatous disease; Aorta, aortogenic embolism; Tro, Trousseau; Dissec, cerebral artery dissection; Other, undetermined etiology; TP, true positives; FN, false negatives; FP, false positives.

### Verification of stroke classification AI

We evaluated the diagnostic accuracy of the stroke classification AI constructed using Cohort 1 using Cohort 2 (total 151 cases). The correct rate for stroke classification (SVO/BAD: 33 cases, CE: 52 cases, LAS: 25 cases, Trousseau syndrome: 9 cases, and ESUS+D: 32 cases) was 86.1% (Tables 6, 7), which was similar to the accuracy rate of Cohort 1 using the LOOCV method.

**Table 6:** Result for stroke classification AI. Matching table for each stroke type; CE, LAS, SVO/BAD, and ESUS+D in Cohort 2.

		Precision subtype by AI				
		CE	LAS	SVO/BAD	Tro	ESUS+D
Manually assigned stroke subtype	CE	**44**	2	4	0	2
	LAS	0	**22**	2	0	1
	SVO/BAD	0	0	**30**	0	2
	Tro	0	0	0	**6**	3
	ESUS+D	2	0	2	0	**28**

Bold numbers indicate the number of each case correctly answered by the AI. Tro, Trousseau syndrome; ESUS + D, embolic stroke of undetermined sources + cerebral artery dissection; TP, true positives; FN, false negatives; FP, false positives.

**Table 7:** Result for stroke classification AI. Precision rate for each stroke classification.

Stroke classification	Patient number TP + FN	Predicted number of cases TP + FP	Number of correct cases TP	Sensitivity TP/(TP + FN)	Precision TP/(TP + FP)	*F* value
CE	52	46	44	85%	96%	0.90
LAS	25	24	22	88%	92%	0.90
SVO/BAD	33	38	30	91%	79%	0.85
Tro	9	7	6	67%	86%	0.75
ESUS+D	32	36	28	88%	78%	0.82

Tro, Trousseau syndrome; ESUS + D, embolic stroke of undetermined sources + cerebral artery dissection; TP, true positives; FN, false negatives; FP, false positives.

## Discussion

In this study, we established SCTSS-AI for cerebral infarction diagnosis that uses image-based infarction detection and medical data to determine the stroke classification. We found that machine learning with the incorporation of clinical information can diagnose cerebral infarction based on the TOAST classification, which could not have been achieved with image-based stroke diagnosis AI alone. The high performance of this SCTSS-AI despite the diversity of MRI images used for construction, and the fact that there is no current AI that can classify the stroke types to provide appropriate treatment according to the initial treatment of cerebral infarction, suggest that this AI can be used in actual clinical practice in the future. SCTSS-AI can make inferences even when some features of data have missing values for a stroke classification, provided that in this verification work, the model was analyzed using cases with no missing data from a single institution. Further studies are needed to investigate whether the model can be analyzed even with missing data, and whether it can be used with high accuracy even when data from multiple institutions are used.

There are different stroke types that require different clinical management. Therefore, classification of stroke types is necessary for early treatment and prevention (23). Subudhi et al. (24), evaluated DWI with a support vector machine classifier according to the Oxfordshire Community Stroke Project (OCSP) classification. They obtained an accuracy of 92.9%, sensitivity of 90.4%, and specificity of 93.3% in differentiating among total anterior circulation infarction, partial anterior circulation infarction, and lacunar circulation infarction. However, posterior circulation infarction, another subclass of OCSP, has not been evaluated, and the MobileNetV2 convolutional neural network (CNN) model, which was fine-tuned to classify cerebral infarcts according to vascular territory, had an accuracy of 93% (25). However, only subtypes covering 75–80% have been evaluated, and this analysis requires the use of a CNN model, which requires a large number of patient cases. The solution to this problem was the creation of the ImageNet dataset, with over 15 million images labeled in 22,000 different categories (26). ImageNet is often used to measure the accuracy of current CNN models. EfficientNet and MobileNetV2 CNN models were preferred for transfer training compared to similar models because of their lower computational load and ImageNet’s higher accuracy (27, 28). However, the advantage of our method is that by inputting MRI, laboratory data, X-ray data, ECG data, and other data used in daily medical treatment of patients into the application, stroke classification can be accurately made, and appropriate treatment can be immediately initiated for patients.

Current AI advancements in stroke TOAST classification are focused on predicting prognosis after the onset of stroke (29). The LAS diagnostic criterion requires over 50% stenosis in proximal arteries (30). A previous study reported the use of computer-based diagnosis utilizing a CNN to identify stenosis (31). In the present study, we aimed to analyze stenosis in MRA using AI. However, AI was considered unsuitable for diagnosis due to instances where the stenotic lesion occluded during the stroke onset; therefore, we employed manual entry. Although this method may miss some cases of stenosis, it was possible to detect stenoses in the ipsilateral carotid artery, internal carotid artery, middle cerebral artery, and basilar artery, and was useful in making a diagnosis. In CE, AI has been developed focusing on the detection of cardiac embolic sources (29). In this study, NT-proBNP was initially considered a potential marker for detecting heart diseases; however, it was deemed unsuitable due to its elevation in patients with chronic kidney disease. Nevertheless, a previous study reported a potential association between NT-proBNP and arrhythmias (32). Further studies with more cases are needed to establish an association curve between eGFR and NT-proBNP, which would make it a suitable candidate for the development of a stroke diagnosis AI. Furthermore, an algorithm attributed to ESUS has been created (33), which may become a candidate in the future. Initially, D-dimer alone was utilized in this study for Trousseau syndrome, but evaluating it was challenging due to the inclusion of many CE cases within the cutoff line. Incorporating data on active malignancy allowed us to achieve a more defined evaluation. Nonetheless, in real clinical scenarios, there are instances where malignancy is unknown at the time of admission, which may impact the accuracy of the response rate.

Although there are limited reports on TOAST classification of ischemic stroke using AI. Primarily, extraction is done from the electronic health records (EHRs), and Garg et al. (34) extracted information from 1,091 cases of EHR data and compared it with actual diagnoses using machine learning techniques. The corresponding precision rates obtained were 70.3% for cardioembolic stroke, 65.3% for large artery atherosclerosis (LAA), 62.3% for small vessel occlusion (SVO), and 73.7% for cryptogenic stroke. Additionally, Zhang et al. (35) performed similar analyzes, resulting in precision rates of 53.3% for cardioembolic stroke, 74.5% for LAA, 54.7% for SVO, and 20.0% for cryptogenic stroke. Moreover, Wang et al. (36) conducted an analysis excluding cryptogenic stroke, achieving precision rates of 94.07% for cardioembolic stroke, 76.73% for LAA, and 72.13% for SVO. However, these reports indicate that our developed system exhibits higher diagnostic accuracy. Furthermore, our system has the advantage of performing analysis inclusive of cryptogenic stroke (also known as ESUS) and incorporating image-based analysis. It is believed that the diagnostic accuracy has improved by creating a two-stage system with ischemia detection AI and subtype classification AI. Furthermore, the comparison registry data, although from a single center, is based on a registry that evaluates cryptogenic stroke with transesophageal echocardiography for aortogenic embolism and stroke associated with patent foramen ovale (PFO). It is considered a strength of the AI developed in this study that it can diagnose detailed subtypes of strokes. SCTSS-AI can change the functional prognosis of stroke patients and lead to the equalization of stroke treatment.

In the future, the use of inflammatory markers and cytokines as predictive elements for AI development should be considered. For example, the ligand for CD40 and expression of MCP1 are upregulated in the acute phase of atherothrombotic stroke, which is also associated with vascular events with diabetes (37). Moreover, the association between white blood cell count and blood glucose at onset and mortality during hospitalization, as well as inflammatory markers are potential factors for stroke diagnostic AI (38). Our findings also suggest that hyperglycemia caused by stroke stress was associated with in-hospital mortality, and there may be a relationship with NO activity. Furthermore, an association between peripheral vasoreactivity index and endothelial function has been reported in the LAS (39). Arterial stiffness indices, such as augmentation index and pulse wave velocity, have been shown to be higher in LAS patients, and arterial stiffness indices at onset may also be useful for the establishment of AI diagnosis in the future.

Our study has several limitations. First, it was a single-center, medical record-based, retrospective study. Second, unlike other AI studies, only a few hundred cases were needed for constructing the AI. Although this was a strength of the study, the small number of cases may also be a limitation. Further studies with more cases may be able to distinguish CE-PFO features and SVO from BAD with only an initial MRI. Third, the features required to distinguish ESUS+D were limited; therefore, the number of cases and characteristics of ESUS+D-determined etiology cases require clarification. Moreover, the diagnosis of cerebral artery dissection requires the collection of vertebral/basilar artery MRA findings. In the present study, the number of cases used for training the AI was insufficient for learning to predict the vascular morphology in areas where no vessels were captured on MRA. Further case collection and prospective randomized studies are needed to address these uncertainties. Fourth, this study had technical limitations. Initially, our proposed method constructed the AI model using 231 cases, which was insufficient to cover all stroke variations. While increasing case numbers may improve our model’s performance, it also introduces exceptions that our model may not classify accurately. This inherent challenge in AI construction is unavoidable. Continuously expanding the dataset and re-building the AI is necessary to yield benefits for patients. Further studies using novel AI models with higher speed are needed to identify exceptions that our model is unable to classify to optimize model structure and hyperparameters.

**Figure 4:**
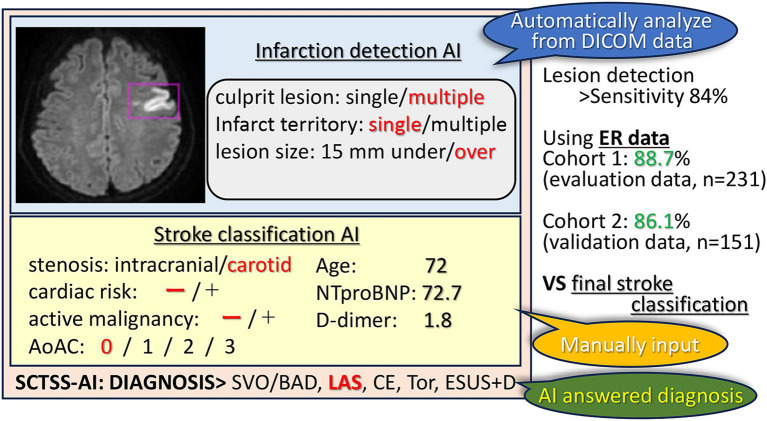
SCTSS-AI concept diagram. The SCTSS-AI identifies and classifies the infarct features (location, number, size) from the DWI on the DICOM data. Although manual input is required, SCTSS-AI automatically determines the type of disease by entering various parameters. Compared to the final stroke classification, the SCTSS-AI provides a definitive diagnosis with an accuracy of more than 86% using data obtained only from the emergency department.

In conclusion, AI in stroke imaging has the potential to revolutionize stroke diagnosis and patient management. Diagnosis of stroke using machine learning methods could be especially useful for health care providers who are not familiar with stroke imaging, such as general practitioners and paramedics, and to speed up treatment decisions. This study, which achieved high accuracy in detecting strokes and classifying their vascular regions, may contribute to the automatic detection of strokes, enabling physicians to make quick and appropriate treatment decisions (Figure 4). Since the AI was created using only factors that are known in the emergency department, we were able to establish an AI that is directly related to clinical practice. Furthermore, our findings suggest that additional tests, such as transesophageal echocardiography and Holter EEG analysis, should be performed if the patient is classified as ESUS+D.

## Data availability statement

The raw data supporting the conclusions of this article will be made available by the authors, without undue reservation.

## Ethics statement

The studies involving humans were approved by the Human Ethics Review Committee of Juntendo University School of Medicine (E22-0028). The studies were conducted in accordance with the local legislation and institutional requirements. The participants provided their written informed consent to participate in this study.

## Author contributions

NM: Conceptualization, Data curation, Formal analysis, Investigation, Methodology, Resources, Validation, Visualization, Writing – original draft, Writing – review & editing. YU: Conceptualization, Data curation, Formal analysis, Methodology, Supervision, Validation, Writing – review & editing. KY: Methodology, Writing – review & editing. KH: Conceptualization, Investigation, Methodology, Writing – review & editing. CK: Investigation, Resources, Writing – review & editing. NK: Investigation, Writing – review & editing. YI: Investigation, Writing – review & editing. KOk: Investigation, Writing – review & editing. SM: Investigation, Writing – review & editing. DT: Writing – review & editing. KOn: Writing – review & editing. MA: Writing – original draft, Writing – review & editing. KM: Writing – review & editing. NH: Supervision, Writing – review & editing.
